# Supplementary material for: Mesenchymal Stem-Cell Remodeling of Adsorbed Type-I Collagen—The Effect of Collagen Oxidation
Source: Int J Mol Sci. 2022 Mar 11;23(6):3058. doi: 10.3390/ijms23063058 (PMC8953637; doi:10.3390/ijms23063058)
Supplement: Supplementary file 1 [file ijms-23-03058-s001.zip › Figure S1.pdf]

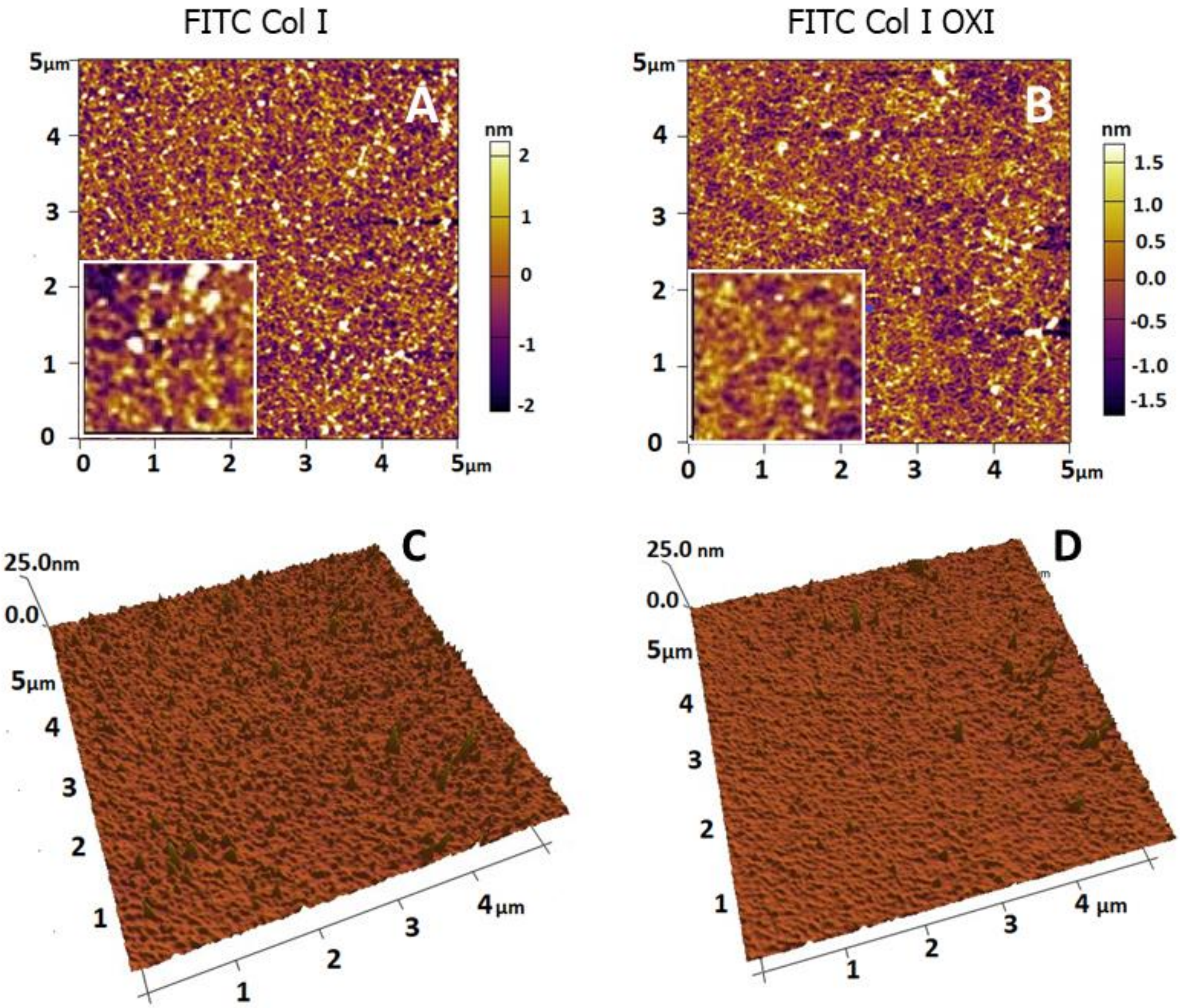

Figure S1. Representative 2D AFM images of native FITC collagen (**A**) and oxidized FITC collagen (**B**); 3D topographical images of the images in A (**C**) and B (**D**). The insets in A and B represent enlarged images of an area with the size of 0.8 × 0.8 µm of the same panel. The AFM imaging was carried out using Atomic Force Microscope Asylum Research MFP-3D (Oxford Instruments) operated in contact mode at room temperature. Silicon nitride probes (Nanosensors, type qp-Bio) with a spring constant of 0.06 N/m, resonant frequency 16 kHz and a nominal tip radius of 8 nm were used in AFM measurements. Morphological characteristics were analyzed using IgorPro software, embedded in the AFM system. The AFM imaging was done in Institute of Optical Materials and Technologies, Bulgarian Academy of Sciences.
