# Supplementary material for: Mesenchymal Stem-Cell Remodeling of Adsorbed Type-I Collagen—The Effect of Collagen Oxidation
Source: Int J Mol Sci. 2022 Mar 11;23(6):3058. doi: 10.3390/ijms23063058 (PMC8953637; doi:10.3390/ijms23063058)
Supplement: Supplementary file 1 [file ijms-23-03058-s001.zip › Table S1.pdf]

Table S1. Quantitative morphological analysis of the % of cells associated with fibrillary arrangements

The cells from altogether 16 samples were counted on the red channel of microscope (viewing actin cytoskeleton) and the percentage of cells that associate with fibrillary arrangements on the underlining FITC-collagen substrate (viewed on the green channel of microscope) was evaluated.

Samples	Number of samples	Average cells per sample<br><br>Mean± SD	Average cells with fibrillary arrangements Mean± SD	Percentage cells with fibrillary arrangements Mean± SD	P
FITC-Col I	6	6.00 ± 1.915	4.83 ± 0.98	85.00 ± 18.03 %	<<0.05
FITC-Col I-OXI	10	6.00 ± 1.09	0.67 ± 0.64	11.22 ± 11.25 %	

*The data were obtained from four separate experiments
