# Supplementary material for: Mesenchymal Stem-Cell Remodeling of Adsorbed Type-I Collagen—The Effect of Collagen Oxidation
Source: Int J Mol Sci. 2022 Mar 11;23(6):3058. doi: 10.3390/ijms23063058 (PMC8953637; doi:10.3390/ijms23063058)
Supplement: Supplementary file 1 [file ijms-23-03058-s001.zip › Table S2.pdf]

Table S2. The efficiency of FITC-collagen binding (F/C)

F/C ratio was calculated from UV-Vis absorption spectra of FITC-Col I and FITC-Col I-OXI (according formula provided in paragraph 4.2 in Material and Methods section).

Collagen Samples	N of samples	Molar F/C	SD	[illegible]
**FITC-Col I**	12	9.365	2.229	> [illegible]
**FITC-Col I-OXI**	12	8.473	2.898	

*The data were obtained from three repeats of quadruplicated samples
