# Supplementary material for: Mesenchymal Stem-Cell Remodeling of Adsorbed Type-I Collagen—The Effect of Collagen Oxidation
Source: Int J Mol Sci. 2022 Mar 11;23(6):3058. doi: 10.3390/ijms23063058 (PMC8953637; doi:10.3390/ijms23063058)
Supplement: Supplementary file 1 [file ijms-23-03058-s001.zip › Table S3.pdf]

Table S3. The efficiency of collagen binding to glass surface

The efficiency was calculated from fluorescence intensity measurement (excitation/emission rate of 485/525 nm) of absorbed FITC-Col I and FITC-Col I-OXI samples on glass bottomed wells. The measurements were done in PBS (pH 7.4) after adsorption performed via incubation of the collagen solutions at concentration 0.10 mg/ml in 0.05M acetic acid for 1h, at 37ºC, followed by 3x washing with PBS.

Collagen Samples	N of samples*	RPU	SD	P
**FITC-Col I**	4	70322.50	4841.97	> 0.05
**FITC-Col I-OXI**	4	62280.25	4398.65	

*The presented data are obtained from a single experiment with quadruplicated samples
